# Alcohol and the Immune System

**Published:** 2015

**Authors:** Dipak Sarkar, M. Katherine Jung, H. Joe Wang

**Affiliations:** Dipak K. Sarkar, Ph.D., D.Phil., is Board of Governors and distinguished professor in the Department of Animal Sciences and director of the Endocrine Program, Rutgers University, New Brunswick, New Jersey. M. Katherine Jung, Ph.D., and H. Joe Wang, Ph.D., are program directors in the Division of Metabolism and Health Effects, National Institute on Alcohol Abuse and Alcoholism, Rockville, Maryland.

Clinicians have long observed an association between excessive alcohol consumption and adverse immune-related health effects such as susceptibility to pneumonia. In recent decades, this association has been expanded to a greater likelihood of acute respiratory stress syndromes (ARDS), sepsis, alcoholic liver disease (ALD), and certain cancers; a higher incidence of postoperative complications; and slower and less complete recovery from infection and physical trauma, including poor wound healing.

This issue of *Alcohol Research: Current Reviews* (*ARCR*) summarizes the evidence that alcohol disrupts immune pathways in complex and seemingly paradoxical ways. These disruptions can impair the body’s ability to defend against infection, contribute to organ damage associated with alcohol consumption, and impede recovery from tissue injury. It is our hope that a greater understanding of the specific mechanisms through which alcohol exerts its effects on the immune system may lead to development of interventions to prevent, or at least mitigate, the negative health consequences of alcohol misuse.

Contributors to this issue of *ARCR* lay the groundwork for understanding the multilayered interactions between alcohol and immune function by presenting an overview of the immune system (see the article by Spiering) and by reviewing current research on the effects of alcohol on innate immunity (see the article by Nagy) and on adaptive immunity (see the article by Pasala and colleagues). As reviewed by Szabo and Saha, alcohol’s combined effects on both innate and adaptive immunity significantly weaken host defenses, predisposing chronic drinkers to a wide range of health problems, including infections and systemic inflammation. Alcohol’s widespread effects on immune function also are underscored in the article by Gauthier, which examines how in utero alcohol exposure interferes with the developing immune system in the fetus. This exposure increases a newborn’s risk of infection and disease; additional evidence suggests that alcohol’s deleterious effects on immune development last into adulthood.

The gastrointestinal (GI) system is typically the first point of contact for alcohol as it passes through the body and is where alcohol is absorbed into the bloodstream. One of the most significant immediate effects of alcohol is that it affects the structure and integrity of the GI tract. For example, alcohol alters the numbers and relative abundances of microbes in the gut microbiome (see the article by Engen and colleagues), an extensive community of microorganisms in the intestine that aid in normal gut function. These organisms affect the maturation and function of the immune system. Alcohol disrupts communication between these organisms and the intestinal immune system. Alcohol consumption also damages epithelial cells, T cells, and neutrophils in the GI system, disrupting gut barrier function and facilitating leakage of microbes into the circulation (see the article by Hammer and colleagues).

These disruptions to the composition of the gut microbiota and to gut barrier function have important implications beyond the intestinal system. For example, Nagy discusses how the leakage of bacterial products from the gut activate the innate immune system in the liver, triggering inflammation that underlies ALD, a condition that affects more than 2 million Americans and which eventually may lead to liver cirrhosis and liver cancer. Infection with viral hepatitis accelerates the progression of ALD, and end-stage liver disease from viral hepatitis, together with ALD, is the main reason for liver transplantations in the United States. The article by Dolganiuc in this issue explores the synergistic effects of alcohol and hepatitis viruses on the progression of liver disease as well as alcohol consumption’s injurious effect on liver antiviral immunity. Mandrekar and Ju contribute an article that homes in on the role of macrophages in ALD development, including recent insights into the origin, heterogeneity, and plasticity of macrophages in liver disease and the signaling mediators involved in their activation and accumulation.

In addition to pneumonia, alcohol consumption has been linked to pulmonary diseases, including tuberculosis, respiratory syncytial virus, and ARDS. Alcohol disrupts ciliary function in the upper airways, impairs the function of immune cells (i.e., alveolar macrophages and neutrophils), and weakens the barrier function of the epithelia in the lower airways (see the article by Simet and Sisson). Often, the alcohol-provoked lung damage goes undetected until a second insult, such as a respiratory infection, leads to more severe lung diseases than those seen in nondrinkers.

In a clinical case study reviewed in this issue, Trevejo-Nunez and colleagues report on systemic and organ-specific immune pathologies often seen in chronic drinkers. In such patients, alcohol impairs mucosal immunity in the gut and lower respiratory system. This impairment can lead to sepsis and pneumonia and also increases the incidence and extent of postoperative complications, including delay in wound closure. HIV/AIDS is a disease in which mucosal immunity already is under attack. Bagby and colleagues review substantial evidence that alcohol further disrupts the immune system, significantly increasing the likelihood of HIV transmission and progression.

Alcohol–immune interactions also may affect the development and progression of certain cancers. Meadows and Zhang discuss specific mechanisms through which alcohol interferes with the body’s immune defense against cancer. They note, too, that a fully functioning immune system is vital to the success of conventional chemotherapy. The clinical management of all of these conditions may be more challenging in individuals who misuse alcohol because of coexisting immune impairment.

Alcohol consumption does not have to be chronic to have negative health consequences. In fact, research shows that acute binge drinking also affects the immune system. There is evidence in a number of physiological systems that binge alcohol intake complicates recovery from physical trauma (see the article by Hammer and colleagues). Molina and colleagues review research showing that alcohol impairs recovery from three types of physical trauma—burn, hemorrhagic shock, and traumatic brain injury—by affecting immune homeostasis. Their article also highlights how the combined effect of alcohol and injury causes greater disruption to immune function than either challenge alone.

Not only does the immune system mediate alcohol-related injury and illness, but a growing body of literature also indicates that immune signaling in the brain may contribute to alcohol use disorder. The article by Crews, Sarkar, and colleagues presents evidence that alcohol results in neuroimmune activation. This may increase alcohol consumption and risky decisionmaking and decrease behavioral flexibility, thereby promoting and sustaining high levels of drinking. They also offer evidence that alcohol-induced neuroimmune activation plays a significant role in neural degeneration and that the neuroendocrine system is involved in controlling alcohol’s effects on peripheral immunity.

Much progress has been made in elucidating the relationship between alcohol consumption and immune function and how this interaction affects human health. Continued advances in this field face several challenges, however. The regulation of immune function is exceedingly complex. Normal immune function hinges on bidirectional communication of immune cells with nonimmune cells at the local level, as well as crosstalk between the brain and the periphery. These different layers of interaction make validation of the mechanisms by which alcohol affects immune function challenging. Significant differences between the immune system of the mouse—the primary model organism used in immune studies—and that of humans also complicate the translation of experimental results from these animals to humans. Moreover, the wide-ranging roles of the immune system present significant challenges for designing interventions that target immune pathways without producing undesirable side effects.

By illuminating the key events and mechanisms of alcohol-induced immune activation or suppression, research is yielding deeper insights into alcohol’s highly variable and sometimes paradoxical influences on immune function. The insights summarized in this issue of *ARCR* present researchers and clinicians with opportunities to devise new interventions or refine existing ones to target the immune system and better manage alcohol-related diseases.

